# Investigating Cognitive Flexibility in Preschool Children With Autism Spectrum Disorder

**DOI:** 10.3389/fpsyg.2021.737631

**Published:** 2021-10-12

**Authors:** Oleg Zacharov, Rene Jürgen Huster, Anett Kaale

**Affiliations:** ^1^Department of Special Needs Education, University of Oslo, Oslo, Norway; ^2^Department of Psychology, University of Oslo, Oslo, Norway; ^3^Norwegian Center of Expertise for Neurodevelopmental Disorders and Hypersomnias, Oslo University Hospital, Oslo, Norway

**Keywords:** cognitive flexibility, preschool children, Autism Spectrum Disorder, typical development, non-verbal mental age

## Abstract

The current study investigated cognitive flexibility in preschool children with Autism Spectrum Disorder (ASD) and those with typical development using the Reverse Categorization (RC) task and the Dimensional Change Card Sort (DCCS) task. We further examined the relationship between non-verbal mental age (NVMA) and the performance on the two tasks. While no significant difference in performance on the RC task between the two groups was found, significantly more children in the typical developing group passed the DCCS task than children in the ASD group. NVMA was found to correlate with performance in both tasks in the typical developing group but not in the ASD group. When the children were matched on NVMA, no differences in task performance between the two groups were found. The current study found the disparity in performance in two groups on the RC and the DCCS tasks, hence illuminating the importance related to the selection of tasks when studying cognitive flexibility in preschool children with ASD. The study also cast some light on the involvement of NVMA in the performance on the RC and DCCS tasks.

## Introduction

Autism Spectrum Disorder (ASD) is characterized by deficits in social communication and interaction, and restricted and repetitive behaviors and interests (American Psychiatric Association, 2013). The term “spectrum” emphasizes that individuals with ASD exhibit wide-ranging levels of symptom severity in language and cognitive functioning. Behavioral difficulties observed in ASD, such as repetitive language and body movements, resistance to change, inflexible thinking, and problems with switching from one activity to another, are all potential indicators of impairment of cognitive flexibility (Smithson et al., 2013).

Cognitive flexibility is one of the major components of executive functioning (EF) and can be described as the ability to switch from one task to another and to quickly adjust to changes in the environment (Diamond, 2013). Cognitive flexibility may be especially important for early academic and social achievements, as it has been shown to correlate with reading comprehension (Cole et al., 2014), abstract mathematics skills (Purpura et al., 2017), and social understanding (Bock et al., 2015).

Difficulties with cognitive flexibility have been documented in persons with ASD on various performance-based EF tasks and rating scales, across different ages and levels of functioning (e.g., Faja and Dawson, 2013; Garon et al., 2017). Children with ASD as young as 3 years were shown to display difficulties on various measurements of cognitive flexibility (Garon et al., 2017). However, the results from studies comparing performance between young (3–7-year-old) typically developing (TD) children and children with ASD on measures of cognitive flexibility are somewhat inconsistent (e.g., Yerys et al., 2006; Gardiner et al., 2017).

For example, Smith et al. (2019) reported similar performance between 1½ and 3-year-old children with ASD and chronological age (CA)-matched TD children on a non-verbal eye-tracking task that assessed cognitive flexibility. However, it should be noted that the ASD group in this study had a significantly lower mental age (MA) and exhibited moderate-to-severe symptoms of ASD. Yerys et al. (2006) also reported no significant differences in performance on cognitive flexibility task between 2 and 3½ year old ASD and TD children matched for MA and CA. Finally, Gardiner et al. (2017) found that performance on task measuring cognitive flexibility, did not differ between 3½ and 7-year-old children with ASD and TD children matched on CA, IQ, and maternal education.

In contrast to the studies reporting null results, Garon et al. (2017) found that 3–6-year-old children with ASD performed worse than MA-matched TD children on the Preschool Executive Functioning Battery (PEFB) measuring, among other things, cognitive flexibility. Also, a study comparing 4–6-year-old children with ASD with CA- and non-verbal IQ (NVIQ)-matched TD children, reported that ASD group exhibited impaired performance (Valeri et al., 2019). Finally, Faja and Dawson (2013) reported that performance of 6–7½ year old children with ASD, as compared to CA and IQ matched TD group, was worse on the task measuring cognitive flexibility.

There are a number of factors that may contribute to the mixed findings within the literature. First, the majority of studies involving young children with ASD employ performance-based EF tasks that may have different levels of difficulty. For each task there is a proposed age at which children with TD are expected to exhibit performance close to or at ceiling. For example, the Reverse Categorization (RC) task (Carlson, 2005), which is purported to measure cognitive flexibility, has been used with TD children between 2 and 4 years. Another measure of cognitive flexibility, the standard Dimensional Change Card Sort (DCCS) task (Zelazo, 2006), has been used with TD children between 2 and 5 years.

Although both the RC and the DCCS are designed to measure cognitive flexibility among preschool children, they may arguably have different difficulty levels. The differences in difficulty level can be attributed to the number of sorting dimensions each task has. The RC task has one sorting dimension, namely color, while the DCCS task has two sorting dimensions, namely color and shape. This may consequentially require more cognitive resources to be deployed in order to perform the DCCS task compared to the RC task (Geurts et al., 2009). On the RC task, TD children have been shown to exhibit near ceiling performance at 3 years of age (Carlson, 2005). On the DCCS task, the majority of 3-year-old TD children usually fail the post-switch condition of the standard version of DCCS, while many 4 and 5-year-old TD children pass it (Zelazo, 2006). This is especially important to consider when interpreting the findings of research in children with ASD. It may be the case that while some EF tasks related to cognitive flexibility may capture the children's impairment, others may not. Hence, it is important to clarify whether preschool children with ASD would elicit differential performance on the RC and DCCS tasks.

Another factor related to the mixed findings is the substantial degree of the heterogeneity of the cognitive profiles in young children with ASD. Due to the inhomogeneous cognitive profiles, it is common to match ASD and TD groups on some general ability, such as IQ or non-verbal mental age (NVMA) measured by standardized tests. Doing so, researchers are controlling for the fact that impaired performance among young children with ASD on EF tasks, including cognitive flexibility, may be a general outcome of developmental delay rather than being specific to the disorder. Although matching ASD and TD children on NVMA is commonplace in studies of EF, the contributions of NVMA on the performance on cognitive flexibility tasks remains under-researched. It has been argued that in TD children the development of cognitive flexibility is strongly associated with verbal development (Karbach and Kray, 2007). Nevertheless, previous research on young TD children has shown that NVMA, measured with the Mullen Scales of Early Learning (MSEL), positively correlated with performance on EF tasks, including cognitive flexibility (Stephens et al., 2018). Since verbal abilities in the majority of ASD population are impaired, some have suggested that cognitive flexibility may not be directly supported by verbal abilities, but instead by NVMA, since ASD individuals seems to rely more on visual rather than verbal abilities when solving EF tasks (Kunda and Goel, 2011). Indeed, in a sample of ASD individuals ranging from 5 to 19 years, Campbell et al. (2017) reported that NVMA, but not verbal mental age, play a unique role in the development of cognitive flexibility. However, the aforementioned studies by Smith et al. (2019) and Yerys et al. (2006) reported similar performance among TD children and children with ASD who had significantly lower NVMA, verbal MA and MA.

In conclusion, there seems to be a lack of research investigating the appropriateness of the cognitive flexibility tasks among preschool children with ASD. Moreover, more knowledge is needed about the relationship between NVMA and the cognitive flexibility performance among preschool children with ASD.

### Rationale

The aim of the current study was to investigate and compare the performance of preschool TD children and children with ASD on two cognitive flexibility tasks, namely the RC and DCCS. Since the two tasks are assumed to have different levels of difficulty, we also examined whether the RC and the DCCS yield similar or contrasting results for a given age group. Although both the RC and the DCCS tasks are designed for preschool children, it is expected that children would struggle more with the DCCS task since it has an extra dimension. Previous research on the appropriateness of executive functioning tasks, including cognitive flexibility, for different age groups were predominantly conducted with typically developing children (Carlson, 2005). Given that impairments in cognitive flexibility are implicated in ASD and the high interest in the topic, it is important that researchers are aware of which tasks may or may not be appropriate for the given study group. The current study would contribute to the field by illuminating the importance of choosing appropriate tasks when studying cognitive flexibility in preschool children with ASD.

In addition to investigating the appropriateness of the tasks, we examined whether there is a relationship between NVMA and the ASD and TD children's performance on the RC and DCCS tasks. In most cases, the NVMA is used as a main matching criterion in studies investigating cognitive flexibility in preschool children with ASD. However, how NVMA is implicated in cognitive flexibility performance among preschool children with ASD remains to be researched. According to previous report by Campbell et al. (2017), it is expected that in the current study the NVMA of preschool children with ASD would be associated with task performance on both tasks.

## Method

### Participants

46 preschool children were recruited for the current study, including 14 children with ASD, aged 40–68 months (M = 56.00, SD = 7.96) and 32 TD children, aged 37–59 months (M = 48.81, SD = 6.95) (Table 1). The ASD group consisted of 12 boys (85.7%) and 2 girls. The TD group consisted of 18 boys (56.3%) and 14 girls.

**Table 1:** Descriptive statistics of typically developing and autism spectrum disorder groups.

	**ASD (*n* = 14)**	**TD (*n* = 32)**	***t***	***p***	**Hedges' *g***
CA (Months)					
M(SD)	56.00 (7.96)	48.81 (6.95)	3.09	0.003	0.99
Range	40–68	37–59			
Social Communication Questionnaire—Parents					
M(SD)	18.85 (6.73)				
Range	8–29				
NVMA (Months)					
M(SD)	32.25 (8.38)	49.13 (9.22)	−5.28	*p* < 0.001	1.88
Range	23–50	29–68			
Receptive language—age (months)					
M(SD)	29.07 (13.33)	51.72 (10.27)	−6.27	*p* < 0.001	2.01
Range	13–62	27–69			
Expressive language —age (months)					
M(SD)	30.14 (17.51)	54.09 (12.69)	−5.23	*p* < 0.001	1.67
Range	14–67	26–70			
Child's spoken language					
Norwegian	8 (57.1%)	15 (46.9%)			
Norwegian + Other	2 (14.3%)	7 (21.9%)			
Missing Data	4 (28.6%)	10 (31.3%)			
Gender					
Male	12 (85.7%)	18 (56.3%)			
Female	2 (14.3%)	14 (43.8%)			
Maternal education					
Primary school	1 (7.1%)				
High school	1 (7.1%)	1 (3.1%)			
University	9 (64.3%)	21 (65.6%)			
Missing data	3 (21.4%)	10 (31.3%)			

Children with ASD were recruited through the specialist health services, educational-psychological services and preschools in Oslo and surrounding counties, while children with TD were recruited through preschools in Oslo and surrounding counties. All children in the ASD group had received a diagnosis of ASD from the specialist health services based on a detailed clinical evaluation including interview with caretakers and multiple observations. All diagnoses were based on the International Classification of Diseases (ICD) 10 (World Health Organization (WHO), 1993). The current study did not validate the children's diagnoses. Instead, the Social Communication Questionnaire (SCQ) (Rutter et al., 2003) were filled out by the parents which informed about the ASD symptoms of the children with ASD. One participant had missing data. Otherwise, all but one child had SCQ scored above the cut-off for ASD (Table 1). In the ASD group, 57,1% of children spoke Norwegian, 14.3% spoke Norwegian and other language and 28.6% had missing data. In the TD group, 46.9% of children spoke Norwegian, 21.9% spoke Norwegian and other language and 31.3% had missing data. Children with severe motor, visual or hearing impairments were not included in the study. The study was approved by Regional Committees for Medical and Health Research Ethics and all parents provided a written informed consent.

### Procedure

The current study was part of a broader longitudinal research investigating early development and learning in children with ASD and TD. All children were administered a number of tests including measures of language and cognitive abilities. Cognitive flexibility was measured with the RC task and the DCCS task. All 32 children in the TD group completed both tasks. In the ASD group, all 14 children completed the RC task, while 13 completed the DCCS task, as one ASD child was excluded from the analysis due to not satisfying the pre-requisite (discussed below) for being scored on the post-switch phase of the DCCS task. The performance on both cognitive flexibility tasks was videotaped. Testing was carried out by the first author and research assistants in a quiet room in the children's preschool or in the laboratory at the University of Oslo. Test duration for each child ranged from 2 to 4 h including breaks. Social (e.g., praise, play brakes) and edible motivators (e.g., raisins, apple bits) were provided when necessary to encourage children to complete the tasks. Demographic information was obtained via questionnaires to parents.

## Measures

### Cognitive and Language Level

The Mullen Scale of Early Learning (MSEL; Mullen, 1995) was used to estimate the children's NVMA and expressive and receptive language level. MSEL is a comprehensive test of language, cognitive and motor functioning that is individually administered to infants and children up to 68 months of age. MSEL consists of five subscales, namely Gross Motor, Fine Motor, Expressive Language, Receptive Language, and Visual Reception. The subscales can be used to calculate an Early Learning Composite Score which is analogous to the traditional IQ score. The Visual Reception and Fine Motor subscales were used to calculate the NVMA, while the Receptive and Expressive subscales were used to calculate language level.

### Executive Functioning Measures

#### Reverse Categorization

RC is purported to measure cognitive flexibility in preschool children (Carlson, 2005). The task requires children to sort objects according to the first rule and then switch to a new sorting rule.

**Figure 1:**
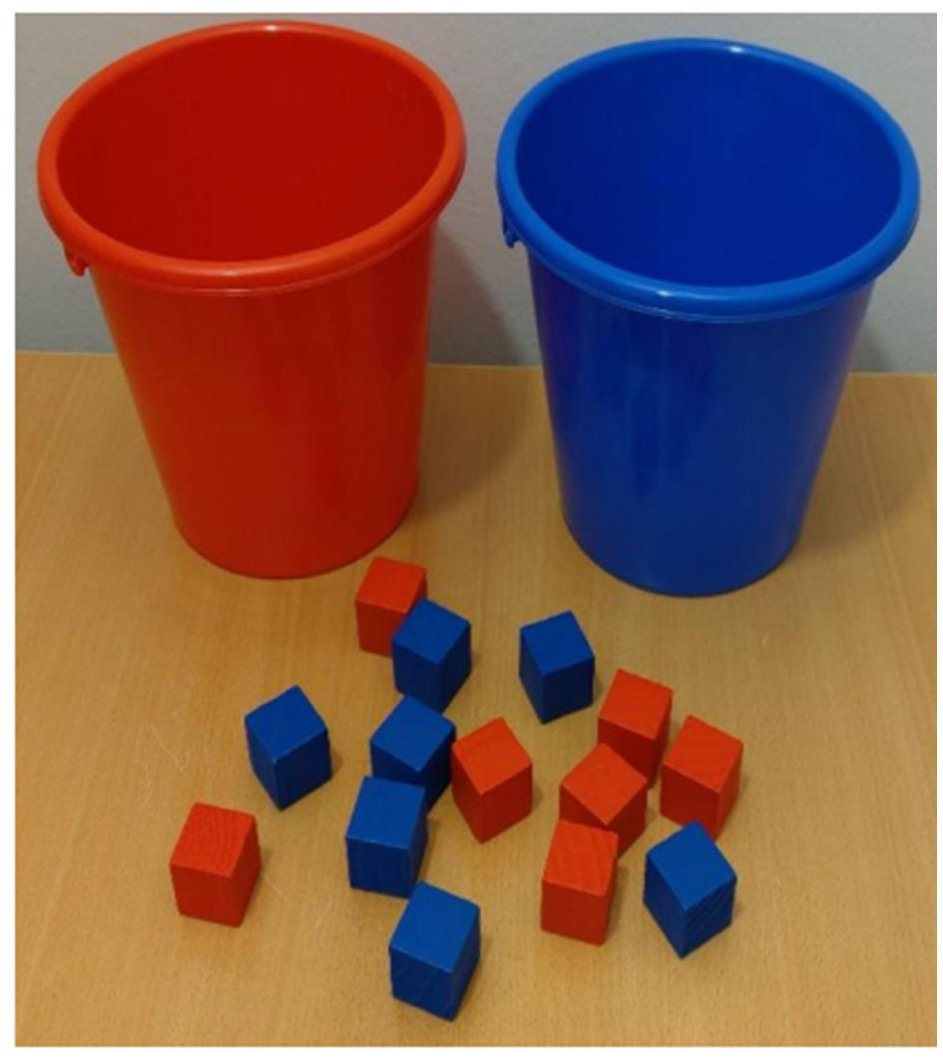
Materials for the Reverse Categorization task.

In the current study, children were presented with a blue and a red bucket that served as sorting containers for 18 blue and 18 red wooden blocks (Figure 1). There were two sorting conditions in this task, namely pre-switch and post-switch conditions. The pre-switch condition required children to put red blocks into a red bucket and blue block into a blue bucket. The post-switch condition required children to put red blocks into a blue bucket and blue blocks into a red bucket. Each sorting condition had 12 trials. Before the administration of the task, children were provided with minimal verbal instructions (“red in red bucket and blue in blue bucket”) and four demonstration blocks (2 red and 2 blue) were sorted by the experimenter. After the demonstration phase, to ensure that the task was understood, children sorted four practice blocks with a rule repeated before every trial. Upon completing the practice session, 12 pre-switch blocks (6 red and 6 blue) were then handed to children one by one in a random order with the rule repeated before every third trial. After completing 12 pre-switch trials, children were informed about the second rule (“red in blue bucket and blue in red bucket”). The experimenter sorted four demonstration blocks while repeating the new rule. After the demonstration phase, no practice session was administered and children were handed 12 post-switch blocks (6 red and 6 blue) one by one in a random order with the rule repeated before every third trial.

Video recordings of children's performance were coded using VLC media player (VideoLan, 2006) and scored by the first author. For each trial the score of 1 was assigned if the child sorted the block according to the rule. The score of 0 was assigned if the child either (1) placed the block into the wrong bucket, or (2) placed the block into the wrong bucket but then took the block out and placed it in the correct bucket. In accordance with established procedures of the task, placing the block in either of the buckets meant the end of the trial, the following actions were disregarded and hence the score of 0 was assigned for the trial in which the child placed a block in a wrong bucket. In order to be scored on post-switch phase of the RC task, children had to correctly sort 10 out of 12 pre-switch trials (Carlson, 2005). The dependent variable was the total number of correctly sorted post-switch trials. This total score was analyzed both categorically, as passing or failing the task, and subjected to Spearman's correlation analyses. In order to pass the task, children were required to sort correctly minimum 10 out of 12 post-switch blocks (Carlson, 2005). For the assessment of inter-rater reliability, a trained research assistant who was blind to the participants' group double-coded 43.48% (*n* = 20) of the RC task recordings. Scoring of the number of sorted pre- and post-switch trials was found to have high reliability (κ = 0.879, 95% CI [0.722–1], *p* < 0.001).

### Dimensional Change Card Sort

DCCS is purported to measure cognitive flexibility (Zelazo, 2006). There are two versions of the DCCS task, namely the standard version, which was used in the current study and the border version which is suitable for older children due to increased complexity. The standard version requires participants to sort a number of cards according to first dimension (e.g., shape), and then according to the second dimension (e.g., color).

**Figure 2:**
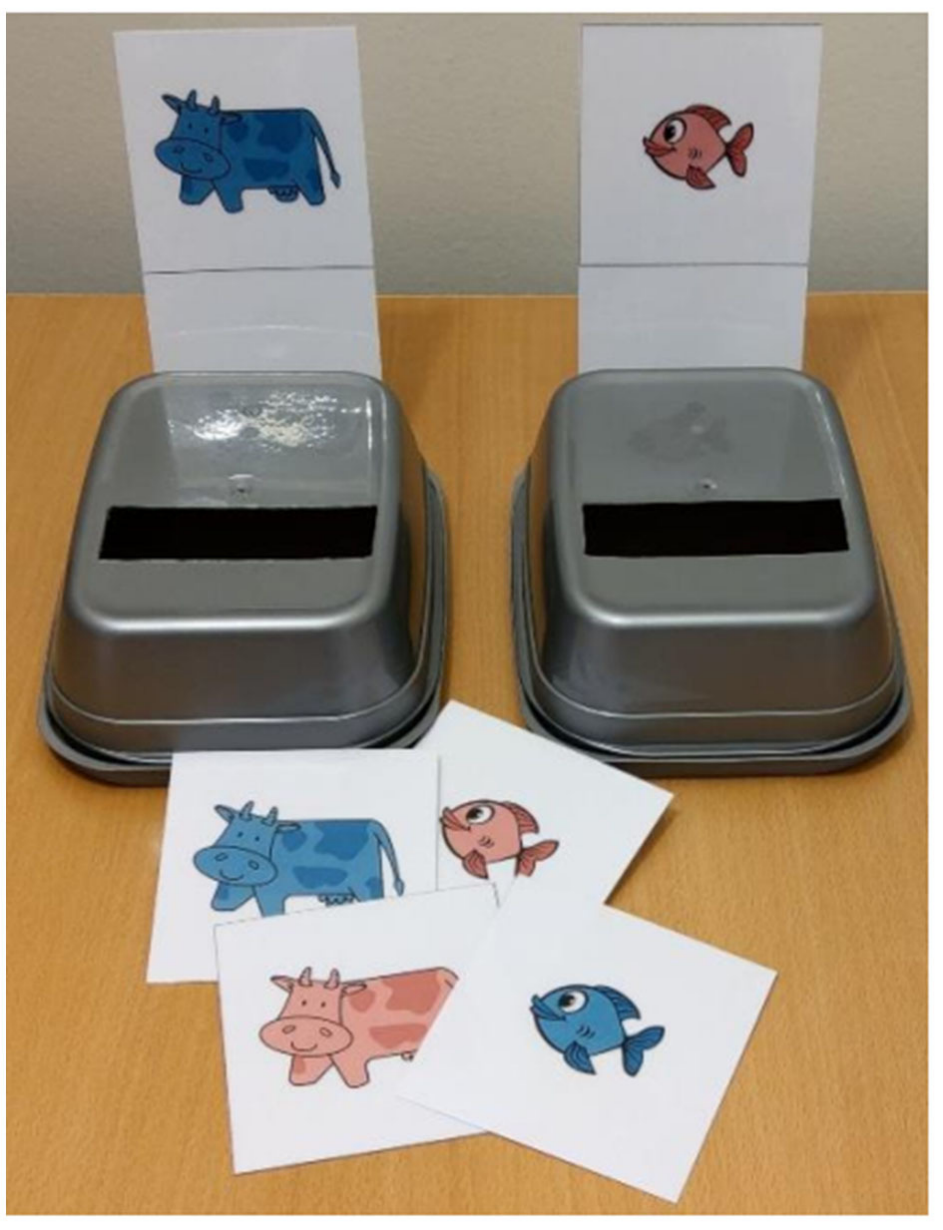
Materials for the Dimensional Change Card Sort task.

In the current study, children were presented with two gray plastic opaque containers. Each container had a slot at the top and a target card (dimensions) depicting either a red fish or a blue cow attached at the back (Figure 2). In total, there were 22 laminated cards depicting blue/red fish and blue/red cow on a white background. Specifically, there were 7 cards depicting a blue fish, 4 depicting a red fish, 4 depicting blue a cow, and 7 depicting a red cow. The task has two sorting conditions: a pre-switch condition, where the cards are sorted according to the first dimension and a post-switch condition, where the cards are sorted according to the second dimension. Each sorting condition had 5 trials. Prior to the experiment, minimal verbal instructions (“red animals go here and blue animals go here” or “fish goes here and cow goes here”) were given and 4 demonstration cards were sorted by the experimenter. To ensure that the task was understood, 4 practice cards were sorted by children with a rule repeated before every trial and each card verbalized by the experimenter. Upon completing the practice session, children were required to sort 5 pre-switch cards. For the “color” dimension, the cards were handed in the following order one by one: blue fish, red cow, red fish, blue fish, and red cow. For the “shape” dimension, the order was as follows: red cow, blue fish, blue cow, red cow, and blue fish. Once all 5 pre-switch cards were sorted, the experimenter informed about the switch (“now we switch”). Children were provided with a new rule and watched the experimenter sort 4 demonstration cards. No practice session was administered for the post-switch condition. Children were handed 5 post-switch cards, one at the time, with the rule repeated before every trial and each card verbalized by the experimenter.

Video recordings of children's performance were coded using VLC media player and scored by the first author. For each trial the score of 1 was assigned if the child sorted the card according to the rule. The score of 0 was assigned if the child placed the card into the wrong container. In order to be scored on post-switch phase of the DCCS task, children had to correctly sort 4 out of 5 pre-switch trials (Zelazo, 2006). Children who did not satisfy this criterion were excluded from the analysis. The dependent variable was the total number of correctly sorted post-switch trials. This total score was analyzed both categorically, as passing or failing, and subjected to Spearman's correlation analyses (Zelazo, 2006). In order to pass the task, children were required to sort correctly minimum 4 out of 5 post-switch cards. For calculation of inter-rater reliability, 43.48% (*n* = 20) of the DCCS task recordings were double-coded by the trained research assistant who was blind to participants' group. Scoring of the number of sorted pre- and post-switch trials was found to have high reliability (κ = 1, *p* < 0.001).

### Statistical Analysis

Statistical Package for the Social Sciences (SPSS) 27 was used to analyze the data. Descriptive data on characteristics (e.g., age, language level, gender) of the ASD and TD groups is presented as means, standard deviations and ranges or frequency and percentages. Independent sample *t*-tests were used to investigate potential group differences in these characteristics. A chi-square test was run separately for the RC and DCCS tasks to investigate whether the number of children passing the task was significantly different between ASD and TD groups and whether the children's performance on the RC and the DCCS were similar or contrasting.

Spearman's correlation analyses were preformed to determine the relationship between NVMA and the total number of correctly sorted post-switch trials on the RC and the DCCS tasks, respectively, for both groups. In these analyses the scores from the RC task and the DCCS task were used as discrete data. Finally, the study participants were matched on NVMA resulting in 9 participants both in the ASD and the TD group (Table 2). A frequency analysis was then run separately for the RC and DCCS tasks to identify number of children passing/failing the tasks in each of the matched groups. Last, Spearman's correlation analyses were run to investigate the relationship between NVMA and the total number of correctly sorted post-switch trials on RC and DCCS for the matched groups.

**Table 2:** Descriptive statistics of typically developing and autism spectrum disorder groups matched on non-verbal mental age.

	**ASD (*n* = 9)**	**TD (*n* = 9)**	***t***	***p***	**Cohen's *d***
CA (Months)					
M(SD)	57.44 (9.06)	44.33 (6.57)	3.51	0.003	1.66
Range	40–68	37–56			
NVMA (Months)					
M(SD)	39.66 (6.47)	41.33 (5.67)	−0.581	0.570	0.27
Range	30–50	31–49			
Receptive language–age equivalent					
M(SD)	36 (11.42)	41.66 (9.04)	−1.16	0.260	0.55
Range	27–62	27–53			
Expressive language —age (months)					
M(SD)	36.88 (18.61)	43.55 (11.08)	−0.923	0.370	0.43
Range	17–67	26–60			

## Results

### Group Differences on the RC and DCCS Tasks

For the TD group, 21 children (65.6%) passed the RC task while 11 (34.4%) did not (Table 3). For the ASD group, 6 children (42.9%) passed the same task and 8 (57.1%) did not. A chi-square analysis revealed no significant differences in performance on the RC task between the groups (*X*^2^ (1, *n* = 46) = 2.08, *p* = 0.149).

**Table 3:** Performance of typically developing and autism spectrum disorder groups on the reverse categorization and the dimensional change card sort tasks.

**Task**	**Group**	
	**ASD (*n* = 14)**	**TD (*n* = 32)**
Reverse categorization		
# and (%) Pass	6 (42.9%)	21 (65.6%)
NVMA—M (SD)	36.50 (7.85)	52.85 (8.17)
# and (%) Fail	8 (57.1%)	11 (34.4%)
NVMA—M (SD)	34.75 (8.19)	43.81 (6.31)
	**ASD (*****n*** **=** **13)**	**TD (*****n*** **=** **32)**
Dimensional change card sort		
# and (%) Pass	2 (15.4%)	17 (53.1%)
NVMA—M (SD)	42.50 (2.82)	54.06 (6.50)
# and (%) Fail	11 (84.6%)	15 (46.9%)
NVMA—M (SD)	35 (7.79)	44.86 (8.34)

*Groups are not matched on non-verbal mental age*.

For the TD group, 17 children (53.1%) passed the DCCS task while 15 (46.9%) did not (Table 3). For the ASD group, only 2 children (15.4%) passed the DCCS task and 11 (84.6%) did not. A chi-square analysis revealed that TD children were significantly more likely than children with ASD to pass the DCCS task (*X*^2^ (1, *n* = 45) = 5.39, *p* < 0.05). For the effect size measure, a Phi Coefficient was run revealing a moderate effect size (φ = 0.346).

### Relation Between NVMA and Performance on the RC and DCCS Tasks

**Figure 3:**
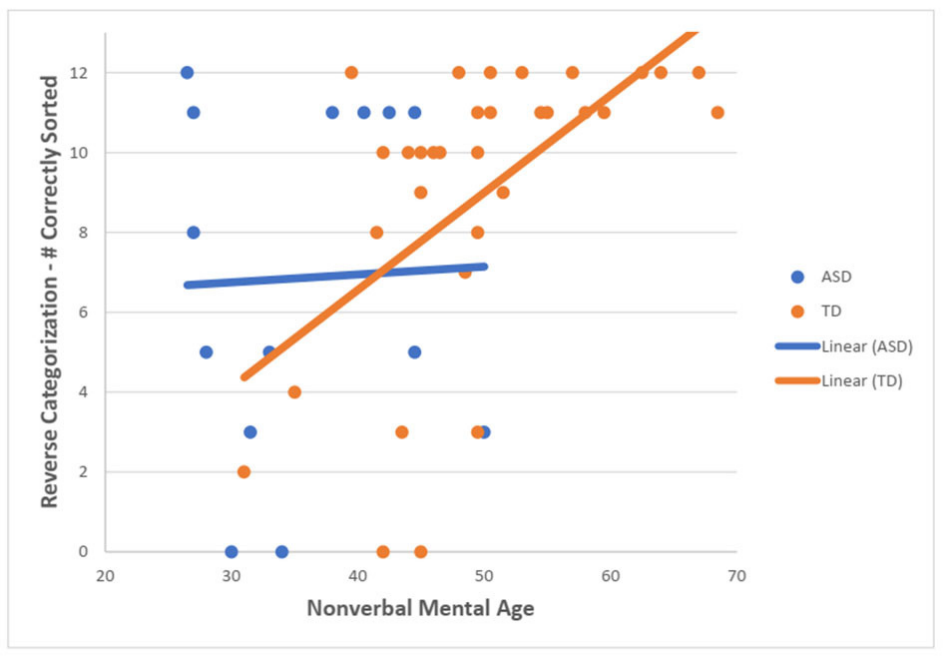
Scotterplot showing the relationship between NVMA and a number of correctly sorted post-switch RC trials for both groups.

For the TD group, there was a statistically significant moderate, positive correlation between NVMA and the total number of correctly sorted post-switch trials on the RC task (r_s_ = 0.635, *N* = 32, *p* < 0.001). For the ASD group, no statistically significant correlation was found (r_s_ = −0.127, *N* = 14, *p* = 0.665) (Figure 3).

**Figure 4:**
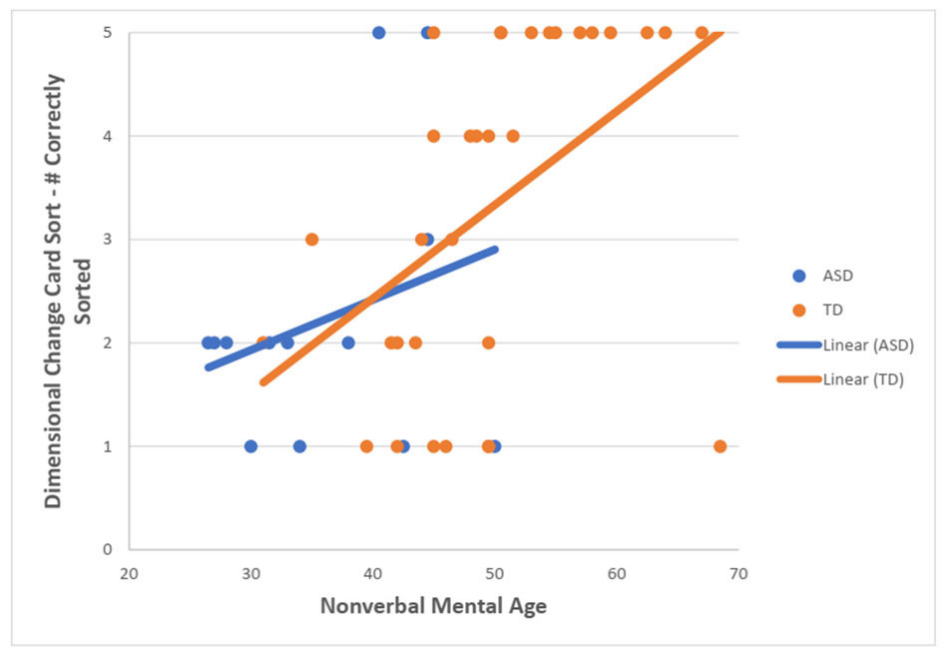
Scatterplot showing the relationship between NVMA and a number of correctly sorted post-switch DCCS trials for both groups.

For the TD group, there was a statistically significant moderate, positive correlation between NVMA and the total number of correctly sorted post-switch trials on the DCCS task (r_s_ = 0.592, *N* = 32, *p* < 0.001). For the ASD group, no statistically significant correlation was found (r_s_ = 0.107, *N* = 13, *p* = 0.727) (Figure 4).

### NVMA—Matched Groups

**Figure 5:**
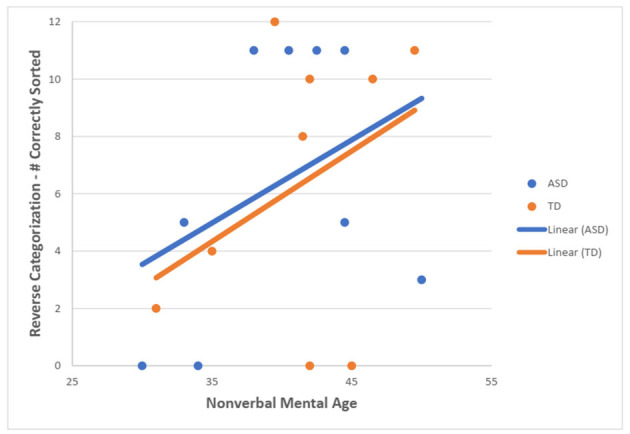
Scatterplot showing the relationship between NVMA and a number of correctly sorted post-switch RC trials for both groups matched on NVMA.

**Figure 6:**
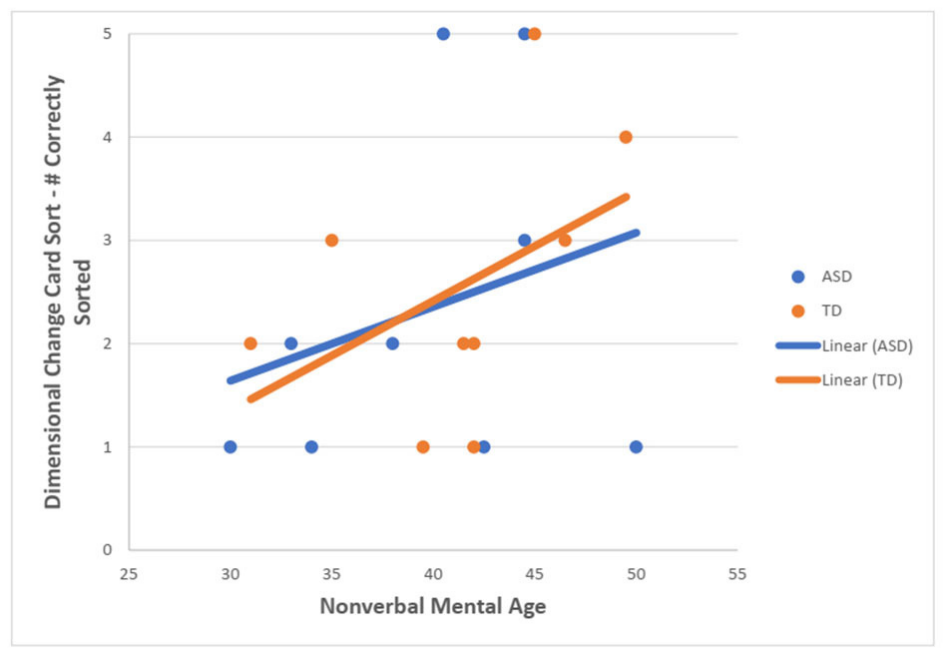
Scatterplot showing the relationship between NVMA and a number of correctly sorted post-switch DCCS trials for both groups matched on NVMA.

The matched groups had equal number of children passing (TD group = 44.4%; ASD group = 44.4%) and failing (TD group = 55.6%; ASD group = 55.6%) the RC task (Table 4). Similarly, both groups had equal number of children passing (TD group = 22.2%; ASD group = 22.2%) and failing (TD group = 77.8%; ASD group = 77.8%) the DCCS task. Finally, as illustrated in Figures 5, 6 neither of the matched groups showed significant correlations between NVMA and RC scores (TD group: r_s_ = 0.194, *p* = 0.617; ASD group: r_s_ = 0.344, *p* = 0.365) or NVMA and DCCS scores (TD group: r_s_ = 0.511, *p* = 0.160; ASD group: r_s_ = 0.251, *p* = 0.514).

**Table 4:** Performance of typically developing and autism spectrum disorder groups on the reverse categorization and the dimensional change card sort tasks.

**Task**	**Group**	
	**ASD (*n* = 9)**	**TD (*n* = 9)**
Reverse categorization		
# and (%) Pass	4 (44.4%)	4 (44.4%)
NVMA—M (SD)	41.37 (2.78)	44.37 (4.47)
# and (%) Fail	5 (55.6%)	5 (55.6%)
NVMA—M (SD)	38.30 (8.52)	38.90 (5.72)
Dimensional change card sort		
# and (%) Pass	2 (22.2%)	2 (22.2%)
NVMA—M (SD)	42.50 (2.82)	47.25 (3.18)
# and (%) Fail	7 (77.8%)	7 (77.8%)
NVMA—M (SD)	38.85 (7.14)	39.64 (5.12)

*Groups are matched on non-verbal mental age*.

## Discussion

The current study investigated whether the performance of preschool children with ASD, as compared to children with TD, was significantly different on two measures of cognitive flexibility, namely the RC task and DCCS task, which have been shown to have different difficulty levels. As for the RC task, which is presumably easier than DCCS task, no statistically significant differences in the number of children passing the task between the two groups were found. In both groups approximately half the children passed the RC task. In contrast, the performance of the ASD group, as compared to the TD group, was significantly lower on the DCCS task. Approximately half the children in the TD group satisfied the passing criterion, while only 15% of the children in the ASD did, despite the fact that the TD children were younger than the ASD group. The results from current study add to the findings of Faja and Dawson (2013) where older children with ASD were shown to exhibit impaired performance on the DCCS task.

Although both the RC and DCCS tasks are developed to measure cognitive flexibility in preschool children (Carlson, 2005) they may reveal different results, not only in children with TD but, as shown in this study, also in children with ASD. We found that slightly more TD children passed the RC task (65.6%) compared to the DCCS (53.1%) task. A similar, but stronger pattern was true for the children with ASD, where almost half passed the RC task (42.9%) and only a few passed DCCS task (15.4%). These results suggest that both tasks, while being designed for preschoolers, challenge children's cognitive flexibility differently. It could be that having two sorting dimension (i.e., color and shape) the DCCS task is more challenging than the RC task because it would presumably require more attentional and\or working memory resources (Geurts et al., 2009). Hence, it may be important to consider the choice of task when studying cognitive flexibility in preschool children, and especially children with ASD. Some tasks may not be suitable for the specific age group or sensitive enough to capture the impairment leading to conclusions that are different from studies using more appropriate measures.

The current study also investigated the relationship between the children's NVMA and their scores on the RC and the DCCS tasks. For the RC task, TD group demonstrated that higher NVMA was correlated with a higher number of correctly sorted post-switch trials. No correlation for the ASD group was found. For the DCCS task, TD children with higher NVMA were more likely to pass the post-switch phase. As for the ASD group, no correlation between NVMA and DCCS scores was found. Previous research demonstrated that most of the TD preschoolers who pass the post-switch condition of DCCS were above 48 months of age (Zelazo, 2006). In the current study, CA and NVMA were shown to strongly correlate in the TD group and the mean NVMA of TD children passing the task was above 48 months while the mean NVMA of TD children who failed the task was below the age of 4. Interestingly, two children in the ASD group who passed the task had mean NVMA of 42.50, which is lower than the level at which children are expected to master the task. Furthermore, the majority of children with ASD who failed the DCCS task, despite having higher CA than TD group, had lower NVMA than those who failed the task in the TD group. Hence, it may be safe to assume that NVMA is implicated in the performance of DCCS. Similarly, NVMA may also be implicated in the RC task as it requires lower levels of NVMA to be able to satisfy the passing criterion, which would also support the notion that RC task is less demanding than DCCS task. Contrary to the Campbell et al. (2017), the findings in the current study cannot suggest that NVMA played a unique role in the cognitive flexibility performance in participants with ASD. While it is true for the TD children, the NVMA had no relationship with the performance on the cognitive flexibility tasks in the ASD group. However, this could be attributed to the small number of participants in the ASD group. With more children in the ASD group, the pattern would maybe be clearer. The current findings could also comment on the study by Yerys et al. (2006) who reported similar performance among TD children and children with ASD who had significantly lower NVMA, verbal MA and MA. As shown in the current study, both groups exhibited similar performance on the RC task while being significantly different in the NVMA. It could be that despite having lower NVMA than the TD group, the ASD group had sufficient NVMA for passing the tasks used in the Yerys et al. (2006) study.

Finally, the current study also matched the groups on NVMA. Although the matched ASD group had significantly higher CA, the performance on RC and DCCS tasks between the two groups were found to be identical. Approximately half the children in each group passed the RC task, while 22.2 percent in both groups passed the DCCS. This further illustrate the possible contributions of NVMA on the performance on measures of cognitive flexibility and that the RC and the DCCS tasks might both be valuable measures of cognitive flexibility in young children with ASD, but that caution is needed in selecting what measure to use. It is important to note, however, that in controlling for NVMA in the matched groups the majority of good-performing TD and a number of bad-performing children with ASD were removed. This would seem to bias the performance measures, despite the fact that children with ASD had a higher CA from the beginning. This would potentially explain the correlation differences seen between NVMA unmatched and NVMA matched groups.

One of the weaknesses of the current study is the small sample size of the ASD group. Hence, caution is needed in interpreting the results. Also due to the small number of children with ASD, and the skewed gender ratio in this population, very few girls were included. Thus, the current study did not investigate potential gender differences in cognitive flexibility although gender differences related to cognitive flexibility have been previously reported (Memari et al., 2013). It is recommended that in future studies investigating cognitive flexibility in preschool children with ASD, sex differences are considered. Despite weaknesses, the current study illuminates some potential problems related to the selection of tasks when studying cognitive flexibility in preschool children with ASD. In future studies, it is recommended to use a broader set of tasks capturing the fine-tuned development of cognitive flexibility during the preschool years. In addition, the study casts some light on the involvement of NVMA in the performance on the RC and DCCS tasks. Given the vast selection of EF tasks, future studies with a larger sample that are matched both on CA and NVMA are needed to investigate tasks that measure different components of EF. There are some clinical implications of the study. First, professionals who want to measure cognitive flexibility in young children with ASD should be critical to what task they use. Cognitive flexibility tasks with less dimensions, such as the RC, might be the first choice for young children with ASD, as tasks with more dimensions, such as DCCS might be too advanced for many. Second, not only the children's chronological age, but also their non-verbal mental age should guide the selection of tasks. Last, as the findings suggest that young children with ASD have more difficulties with cognitive flexibility compared to TD peers, whether these difficulties are related to ASD or more general developmental delay, it is important to adapt the early education setting to accommodate these difficulties.

## Data Availability Statement

The data analyzed in this study is subject to the following licenses/restrictions: Not available due to restrictions related to ethical regulations. Requests to access these datasets should be directed to Anett Kaale, anett.kaale@isp.uio.no.

## Ethics Statement

The current study was reviewed and approved by Regional Committees for Medical and Health Research Ethics. Written informed consent to participate in this study was provided by the participants' legal guardian/next of kin.

## Author Contributions

The data collection in the current study was conducted by OZ. All authors contributed in writing the manuscript and took part in the design and analyses of this study.

## Funding

This research was supported by grants from Department of Special Needs Education at University of Oslo, Norwegian Center of Expertise for Neurodevelopmental Disorders and Hypersomnias at Oslo University Hospital, and the research group Communicative Processes (ComPros) at Department of Special Needs Education at University of Oslo.

## Conflict of Interest

The authors declare that the research was conducted in the absence of any commercial or financial relationships that could be construed as a potential conflict of interest.

## Publisher's Note

All claims expressed in this article are solely those of the authors and do not necessarily represent those of their affiliated organizations, or those of the publisher, the editors and the reviewers. Any product that may be evaluated in this article, or claim that may be made by its manufacturer, is not guaranteed or endorsed by the publisher.
